# Dietary and Health Characteristics of Korean Adults According to the Level of Energy Intake from Carbohydrate: Analysis of the 7th (2016–2017) Korea National Health and Nutrition Examination Survey Data

**DOI:** 10.3390/nu12020429

**Published:** 2020-02-07

**Authors:** Sue Min Soh, Sang-Jin Chung, Jihyun Yoon

**Affiliations:** 1 Department of Food and Nutrition, Seoul National University, Seoul 08826, Korea; pa2007@snu.ac.kr; 2 Department of Foods and Nutrition, Kookmin University, Seoul 02707, Korea; schung@kookmin.ac.kr

**Keywords:** KNHANES, carbohydrate intake, energy intake from carbohydrate

## Abstract

The purpose of this study was to examine the association between the level of energy intake from carbohydrate and the dietary and health characteristics among Korean adults. We examined the diet quality and health conditions of Korean adults by segmenting them into eight groups according to the level of energy intake from carbohydrate (<45%, 45–50%, 50–55%, 55–60%, 60–65%, 65–70%, 70–75%, and ≥75%). From the data of the 7th (2016–2017) Korea National Health and Nutrition Examination Survey (KNHANES), 7566 subjects aged 19 to 64 years were analyzed. Diet quality was much lower in the groups whose energy intake from carbohydrate was <50% or ≥65%, compared to the groups whose energy intake from carbohydrate was 50–65%. Hypertension or low HDL-cholesterolemia was associated with low (<45%) or high (≥70%) energy intake from carbohydrate. We found no considerable difference in the diet quality and health conditions between the groups whose energy intake from carbohydrate was 50–55% and 55–65%. In conclusion, it is suggested to expand the current acceptable macronutrient distribution range (AMDR) for carbohydrate for Korean adults (i.e., 55% to 65%) to include 50–55%.

## 1. Introduction

As the intake of animal-source food has increased, energy intake from carbohydrate has continuously decreased among Koreans. The mean of energy intake from carbohydrate of Koreans decreased from 80.3% in 1969 to 63.6% in 2017 [1,2]. For the last decade, the proportion of Koreans with less than 55% of their energy intake from carbohydrate has increased, while the proportion of those with greater than or equal to 70% of their energy intake from carbohydrate has decreased. As carbohydrate intake recently has become the focus of public attention, low-carbohydrate and high-fat diets which increase fat intake with reducing carbohydrate intake are prevailing for weight loss among some Korean adults. This accelerates the decrease of the energy intake from carbohydrate among Koreans, especially young adults.

According to the Dietary Reference Intakes for Koreans 2015, the current acceptable macronutrient distribution range (AMDR) for carbohydrate is 55 to 65% for Korean adults [3]. The former AMDR for carbohydrate, which was 55 to 70% for Korean adults, was revised to 55 to 65% due to a negative health impact of high carbohydrate intake. The lower limit of the current standard of energy intake from carbohydrate for adults is higher in Korea compared to the United States, Japan, China, and the United Kingdom; the current AMDR for carbohydrate is 45 to 65% in the United States, 50 to 65% in Japan and China, and 50% in the United Kingdom for adults [4,5,6,7]. Several studies have reported that dietary or health characteristics might be different according to the level of energy intake from carbohydrate [8,9,10,11,12,13]. The intakes of protein, fat, thiamine, riboflavin, niacin, calcium, phosphorus, and iron were significantly lower in the group whose energy intake from carbohydrate was >70%, compared to the group whose energy intake from carbohydrate was 55–70% [8]. In another study, the higher the level of energy intake from carbohydrate, the greater the increase in the number of servings of grains and fruits and the greater the decrease in the number of servings of meat/fish/eggs/legumes and dairy products [9]. Eating above a certain level of carbohydrate has a negative effect on health. For example, as the energy intake from carbohydrate increases, the HDL-cholesterol concentration tends to decrease [10,11]. A high carbohydrate intake is associated with metabolic syndrome [11,12,13].

Although we found a few studies with low carbohydrate intake [8,12], there has been very limited research on energy intake less than 55% from carbohydrate in Asian population. Moreover, the group with energy intake less than 55% from carbohydrate was not further segmented, but rather regarded as a single group [9,14,15]. As the proportion of Korean adults with energy intake less than 55% from carbohydrate has increased, we examined the diet quality and health conditions of Korean adults by segmenting them according to their level of energy intake from carbohydrate. Especially, we further segmented the group with energy intake less than 55% from carbohydrate.

## 2. Materials and Methods

### 2.1. Data Source and Subjects

We used the data from the 7th (2016–2017) Korea National Health and Nutrition Examination Survey (KNHANES). KNHANES is a cross-sectional and nationally representative survey that uses a stratified, multistage sampling method designed for selection of household units. It is administered by the Korea Center for Disease Control and Prevention to identify Korean health and nutrition status. The survey is comprised of three sections: health interview, health examination, and nutrition. The health interview survey and the health examination survey are conducted at the medical check-up vehicles that move around survey areas. The nutrition survey is performed by experienced nutritionists using a one-day 24-h recall method to investigate each respondent’s food intake, eating behavior, and food security [16].

Among the 9597 eligible subjects aged 19 to 64 years, the subjects who did not respond to the health interview survey, the health examination survey, or the nutrition survey, and the subjects who reported implausible energy intake (<500 or >5000 kcal/day) were excluded. Also, a subject who consumed nothing but alcohol was excluded. Consequently, a total of 7566 adults were included in this study and segmented into eight groups by level of energy intake from carbohydrate: less than 45% (<45%), 45% to less than 50% (45–50%), 50% to less than 55% (50–55%), 55% to less than 60% (55–60%), 60% to less than 65% (60–65%), 65% to less than 70% (65–70%), 70% to less than 75% (70–75%), and 75% or more (≥75%). In this study, the level of energy intake from carbohydrate was calculated as the percentage of calories from carbohydrate intake out of the total calorie intake.

### 2.2. Assessment of Dietary Characteristics

To assess nutrient adequacy, the nutrient adequacy ratio (NAR) was calculated for protein and eight micronutrients (vitamin A, thiamin, riboflavin, niacin, vitamin C, calcium, phosphorus, and iron). For a given nutrient, the NAR is the ratio of a subject’s intake to the recommended nutrient intake (RNI), but limited not to exceed 1. The nutrient consumed is less than the RNI when the NAR is <1; the nutrient consumed is greater than or equal to RNI when the NAR is 1 [17]. To evaluate the overall nutrient intake, the mean adequacy ratio (MAR), which is the mean value of nine NARs, was calculated. A cut-off point of 0.75 was used for NAR and MAR to evaluate the adequacy of nutrient intake [18,19,20,21]. The prevalence of subjects who consumed less than the estimated average requirement (EAR) was calculated for protein and seven micronutrients (vitamin A, thiamin, riboflavin, niacin, vitamin C, calcium, and iron). In this study, assessment of nutrient intake was performed based on the Dietary Reference Intakes for Koreans 2015 [3]. To assess the food group intake, the dietary diversity score (DDS) and the food group intake patterns were computed based on the method reported by Kant et al. [22,23]. Food items were classified into five major food groups: grains, meat/fish/eggs/legumes, vegetables, fruits, and dairy products. The DDS is the sum of each food group’s points, and the food group intake patterns are a combination of a five-digit number with 0 or 1 for each of the food groups in the order of dairy products, meat/fish/eggs/legumes, grains, fruits and vegetables (DMGFV). For each food group, 1 was assigned when more than the minimum amount of a food group was consumed; otherwise, 0 was assigned. For the dairy products and grains, the minimum amount is 15 g for all solids and 30 g for all liquids. For meat/fish/eggs/legumes, fruits, and vegetables, the minimum amount is 30 g for all solids and 60 g for all liquids. 

### 2.3. Assessment of Health Characteristics

We examined the associations of the obesity, hypercholesterolemia, metabolic syndrome, or risk factors of metabolic syndrome with the level of energy intake from carbohydrate. The obesity or hypercholesterolemia criteria were based on the Guidelines for the use of raw data of the 7th (2016–2017) Korea National Health and Nutrition Examination Survey [16]. Metabolic syndrome was classified according to the diagnosis criteria presented in the National Cholesterol Education Program Adult Treatment Panel III [24], with modification of waist circumference cutoff due to the subjects being Korean [25]. Metabolic syndrome was defined if three or more of the following items were present: (1) waist circumference: greater than or equal to 90 cm for men, greater than or equal to 85 cm for women; (2) triglycerides: greater than or equal to 150 mg/dL or in the treatment of related medication; (3) HDL-cholesterol: less than 40 mg/dL for men, less than 50 mg/dL for women; (4) blood pressure: systolic blood pressure greater than or equal to 130 mmHg, diastolic blood pressure greater than or equal to 85 mmHg, or in the treatment of related medication; and (5) fasting glucose: greater than or equal to 110 mg/dL or in the treatment of related medication.

### 2.4. Statistical Analysis

All statistical analyses were performed using SPSS (version 23.0; IBM Corp., Armonk, NY, USA). As recommended by the Korea Centers for Disease Control and Prevention, this study was performed with a complex sample module including a stratification variable, a clustering variable, and a weight variable [26]. The results are reported as weighted percentage, mean values, and standard errors (SE). The difference between eight groups was tested for statistical significance using Rao–Scott χ^2^ test or analysis of covariance (ANCOVA) with sex, age, and total energy intake as covariates. Multiple logistic regression was conducted to test association between the level of energy intake from carbohydrate and health conditions. We chose a group whose energy intake from carbohydrate was 60–65% as a reference for the analysis. A *p*-value < 0.05 was regarded an indication of statistical significance.

## 3. Results

### 3.1. Proportion of Subjects According to the Level of Energy Intake from Carbohydrate

**Figure 1:**
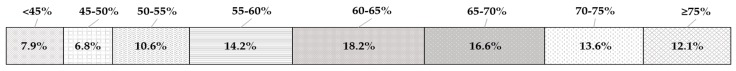
Proportion of Korean adults aged 19 to 64 years according to the level of energy intake from carbohydrate (*n* = 7566). Note: The data were analyzed using the complex sample module. <45%: less than 45%, 45–50%: 45% to less than 50%, 50–55%: 50% to less than 55%, 55–60%: 55% to less than 60%, 60–65%: 60% to less than 65%, 65–70%: 65% to less than 70%, 70–75%: 70% to less than 75%, and >75%: 75% or more.

Figure 1 shows the proportion of subjects according to the level of energy intake from carbohydrate. The highest percentage of subjects, which was 18.2%, was in the group of 60–65% energy intake from carbohydrate. About 25% of subjects was in the groups with energy intake less than 55% from carbohydrate and about 42% of those were in the groups whose energy intake from carbohydrate was 65% or more.

### 3.2. Demographic Characteristics of Subjects

Table 1 shows the demographic characteristics of the subjects according to the level of energy intake from carbohydrate. The distribution of sex (*p* < 0.001), age (*p* < 0.001), residential area (*p* = 0.001), household income level (*p* < 0.001), education level (*p* < 0.001), employment status (*p* = 0.017), marriage status (*p* < 0.001), and the number of family members (*p* < 0.001) were significantly different among the eight groups by the level of energy intake from carbohydrate.

Unlike the other groups, the percentage of women was higher than that of men in the groups whose energy intake was 70–75% or ≥ 75%. Subjects aged 19 to 29 years were more likely to have lower carbohydrate intake, whereas those aged 50 to 64 years were more likely to have higher carbohydrate intake. The percentage of those with higher household income or with higher than college graduate showed lower energy intake from carbohydrate.

### 3.3. Dietary Characteristics

Table 2 shows the NARs and MARs of Korean adults according to the level of energy intake from carbohydrate. The NARs of protein (*p* < 0.001), vitamin A (*p* < 0.001), thiamin (*p* < 0.001), riboflavin (*p* < 0.001), niacin (*p* < 0.001), vitamin C (*p* < 0.001), calcium (*p* < 0.001), phosphorus (*p* < 0.001), iron (*p* = 0.022), and MARs (*p* < 0.001) were significantly different among the eight groups. The NARs of vitamin A, vitamin C, and calcium were below 0.75 in all groups. The NARs of riboflavin and niacin were below 0.75 only in the group whose energy intake from carbohydrate was ≥75%. The MAR was below 0.75 only in the groups whose energy intake from carbohydrate was 70–75% or ≥75%.

The NARs of vitamin A and calcium were lowest in the group whose energy intake from carbohydrate was ≥75%, and the NAR of vitamin C was lowest in the group whose energy intake from carbohydrate was <45%. The MAR was lowest in the group whose energy intake from carbohydrate was ≥75%, followed by the group of 70–75% energy intake from carbohydrate.

**Figure 2:**
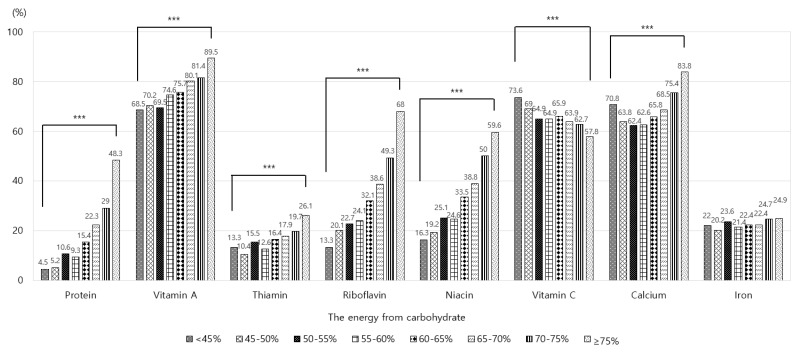
Percentage of Korean adults with insufficient nutrient intakes according to the level of energy intake from carbohydrate. Note: The data were analyzed using the complex sample module. <45%: less than 45%, 45–50%: 45% to less than 50%, 50–55%: 50% to less than 55%, 55–60%: 55% to less than 60%, 60–65%: 60% to less than 65%, 65–70%: 65% to less than 70%, 70–75%: 70% to less than 75%, and >75%: 75% or more. The values are the percentages of those who consumed nutrients less than estimated average requirements. *** Significantly different between eight groups at *p* < 0.001 by Rao–Scott χ^2^ test.

Figure 2 shows the prevalence of Korean adults who consumed less than the EAR of protein and selected micronutrients according to the level of energy intake from carbohydrate. The prevalence of adults with insufficient intakes of protein, vitamin A, thiamin, riboflavin, niacin, vitamin C, and calcium was significantly different among the eight groups (*p* < 0.001), but this was not the case for iron. The prevalence of those with insufficient intake of vitamin A, vitamin C, or calcium was higher than 50% in all groups. The prevalence of adults with insufficient intake of vitamin A or calcium was highest in the group whose energy intake from carbohydrate was ≥75%, and those with insufficient intake of vitamin C was highest in the group whose energy intake from carbohydrate was <45%. The prevalence of adults with insufficient intake of other nutrients such as protein, thiamin, riboflavin, and niacin tended to increase as the level of energy intake from carbohydrate increased, and it was highest in the group whose energy intake from carbohydrate was ≥75%.

Table 3 shows the DDS and its distribution for Korean adults according to the level of energy intake from carbohydrate. The DDS was significantly different among the eight groups (*p* < 0.001). The DDS was lowest in the group whose energy intake from carbohydrate was ≥75%. The DDS was significantly lower in the groups whose energy intake from carbohydrate was <45% or ≥75%, compared to the groups whose energy intake from carbohydrate was 50–55%, 55–60%, 60–65%, or 65–70%. The group of 45–50% energy intake from carbohydrate showed a lower DDS compared to the group of 55–60% energy intake from carbohydrate.

The group whose energy intake from carbohydrate was 70–75% showed a lower DDS compared to the two groups whose energy intake from carbohydrate was 55–60% or 60–65%. As the level of energy intake from carbohydrate increased, the DDS tended to increase until the energy intake from carbohydrate was 55–60% and 60–65%, whereas it tended to decrease after those levels. The distribution of DDS was significantly different among the eight groups (*p* < 0.001). The percentage of adults with DDS of 5 was much lower in the groups whose energy intake from carbohydrate was <50% or ≥75%, compared to the others.

Table 4 shows the food group intake patterns in Korean adults according to the level of energy intake from carbohydrate. The food group intake patterns were significantly different among the eight groups (*p* < 0.001). The percentage of those who consumed less than minimum amount of both dairy products and fruits was much higher in the groups whose energy intake from carbohydrate was <45% or 45–50%, compared to the others. The percentage of those who consumed less than the minimum amount of dairy products was much higher in the groups whose energy intake from carbohydrate was 65–70%, 70–75% and ≥75% compared to the others, which was about 42% of subjects in the group of ≥75% energy intake from carbohydrate. The nutrient and food group intakes of Korean adults according to the level of energy intake from carbohydrate are presented in Tables S1 and S2, respectively.

### 3.4. Health Characteristics

Table 5 shows the association of the energy intake from carbohydrate with obesity, hypercholesterolemia, metabolic syndrome or risk factors of metabolic syndrome among Korean adults after adjusting for age, sex and total energy intake. For low HDL-cholesterolemia, the group whose energy intake from carbohydrate was ≥75% showed an OR of 1.37 (95% CI, 1.10–1.70). For hypertension, the group whose energy intake from carbohydrate was <45% showed an OR of 1.48 (1.10–2.00) and the group whose energy intake from carbohydrate was 70–75% showed an OR of 1.25 (1.00–1.55). However, the associations of the energy intake from carbohydrate with obesity, hypercholesterolemia, metabolic syndrome, high waist circumference, hypertriglyceridemia, or impaired fasting glucose were not significant. The blood biochemical indices of Korean adults according to the level of energy intake from carbohydrate are presented in Table S3.

## 4. Discussion

The current AMDR of energy intake from carbohydrate for adults is 55 to 65% in Korea, 50 to 65% in Japan, 45 to 65% in the United States, and 50% in the United Kingdom [3,4,5,7]. The mean energy intake from carbohydrate among adults is higher in Korea compared to the other countries mentioned above; it was 64.0% in Korea, 57.7% in Japan, 47.0% in the United States, and 45.7% in the United Kingdom [2,27,28,29]. However, carbohydrate intake has been decreased dramatically among Koreans due to westernized diet, which was related to increase the risk of being overweight or obesity [30,31]. In reality, our study showed that the percentage of those with energy intake less than 55% from carbohydrate was 25.3%, which is more than six times higher than 11 years ago. A study using the data from the 3rd (2005) KNHANES reported that the percentage of those with energy intake less than 55% from carbohydrate among subjects aged 20 to 69 years was 4.1% [14]. Therefore, it would be necessary to identify appropriate AMDR that reflects current carbohydrate intake of Koreans.

We examined the association of diet quality and health conditions with the level of energy intake from carbohydrate, and found that we could suggest possibly to add 50–55% energy intake from carbohydrate as revised AMDR into current one for carbohydrate in Korean adults. In this study, we further segmented categories of carbohydrate intake according to the level of energy intake from carbohydrate (<45%, 45–50%, 50–55%, 55–60%, 60–65%, 65–70%, 70–75%, and ≥75%) to explore the possibility of AMDR expansion for carbohydrate in Korean adults.

Recently, some studies have reported that high and low energy intake from carbohydrate is associated with the high mortality [32,33]. According to a study which analyzed the data from the Atherosclerosis Risk in Communities (ARIC) study, there was a U-shaped association of energy intake from carbohydrate and mortality, and the risk of mortality was lowest when energy intake from carbohydrate was 50% to 55%, which implies that both high and low energy intake from carbohydrate is associated with increased mortality [32]. Our results also showed that hypertension was more likely to be present at both high (70–75%) and low (<45%) energy intake from carbohydrate, and low HDL-cholesterolemia was more likely to be present at high (≥75%) energy intake from carbohydrate, compared to the reference group whose energy intake from carbohydrate was 60–65%.

Many studies have examined the relationship between carbohydrate intake and obesity, metabolic syndrome, hypertriglyceridemia, low HDL-cholesterolemia, hypertension, or impaired fasting glucose. In accordance with our study, a high carbohydrate intake was associated with low HDL-cholesterolemia in the previous studies [11,12,14,34]. A study with subjects aged 19 years or older reported that the odds ratio for low HDL-cholesterolemia of those in the highest quintile of energy intake from carbohydrate was 1.31 in men and 1.45 in women compared to the lowest quintile of energy intake from carbohydrate [11]. Another study including subjects aged 40 to 69 years reported that the odds ratio for low HDL-cholesterolemia of those in the highest quartile of the energy intake from carbohydrate was 1.23 compared to the lowest quartile of energy intake from carbohydrate [12].

Several studies reported that high carbohydrate intake was associated with metabolic syndrome or its risk factors, such as hypertriglyceridemia in men or women [9,11,12,13,15,34,35,36]. Obesity was also reported to be associated with high carbohydrate intake due to continued elevation of blood insulin [37]. However, the link between carbohydrate intake and hypertension or impaired fasting glucose was unclear [8,9,11,14,38]. Being hypertensive with high carbohydrate intake in our results may be associated with high sodium intake by fermented vegetable in Koreans’ traditional carbohydrate-rich diet or kidney related mechanisms by insufficient vitamin A consumption, not with carbohydrate intakes themselves [39,40]. We did not find the association between carbohydrate intake and obesity, hypercholesterolemia or metabolic syndrome. However, this might be due to using moderate carbohydrate intake (60–65% energy intake from carbohydrate) as a reference to compare groups in our study, whereas most studies used lowest or highest quartile as a reference. Further studies are needed to clarify the relation of the carbohydrate intake to the aforementioned risk factors.

In our study, the MAR was below 0.75 in the groups whose energy intake from carbohydrate was ≥70%, indicating that overall nutrient intake was inadequate. The NARs of vitamin A and calcium were lowest in the group whose energy intake from carbohydrate was ≥75%, and the NAR of vitamin C was lowest in the group whose energy intake from carbohydrate was <45%. These results are similar with a study which segmented the subjects into two groups according to the level of energy intake from carbohydrate, 55–70% and >70% [8]. According to that study, the MAR was below 0.75 only in the group whose energy intake from carbohydrate was >70%. The NARs of vitamin A, riboflavin, vitamin C, and calcium were below 0.75 in both the groups. Also, the NARs of vitamin A, thiamin, riboflavin, niacin, and calcium were significantly lower in the group whose energy intake from carbohydrate was >70%, compared to the other group. In summary, the NARs of some selected nutrients or the MAR were lower in the groups whose energy intake from carbohydrate was <45% or ≥70%, compared to the others in our study. The reason for the difference of MARs among eight groups could be due to the different micronutrients intakes by changes of the level of energy intake from protein or fat, which was affected by various levels of energy intake from carbohydrate.

In the present study, the prevalence of those who consumed less than the EAR for each of vitamin A, vitamin C, and calcium was more than 50% in all groups. This indicates that many Korean adults inadequately consumed those nutrients regardless of the level of energy intake from carbohydrate. Above all, the prevalence of those with insufficient intake of vitamin A or calcium was highest in the group whose energy intake from carbohydrate was ≥75%, and those with insufficient intake of vitamin C was highest in the group whose energy intake from carbohydrate was <45%. Meat/fish/eggs/legumes intake has been reported to have a 15% or more contribution to vitamin A intake in the Korean population [41], and it is inversely associated with the carbohydrate intake [9]. Therefore, this could be the reason why the prevalence of those with insufficient intake of vitamin A tended to increase as the level of energy intake from carbohydrate increased. Vitamin C is mostly consumed by eating vegetables or fruits and the major sources of calcium are dairy products in Korea [3]. A study which analyzed the data from the 6th (2013–2015) KNHANES reported that the number of servings of fruits tended to increase whereas the number of servings of dairy products tended to decrease as the level of energy intake from carbohydrate increased [9]. Therefore, this might be the reason why the prevalence of those with insufficient intake of vitamin C tended to decrease and that of calcium tended to increase as the level of energy intake from carbohydrate increased. In summary, the prevalence of those who inadequately consumed vitamin A, vitamin C, or calcium was highest at either the group whose energy intake from carbohydrate was <45% or the group whose energy intake from carbohydrate was ≥75%.

The percentage of subjects with a DDS of 5 in our study was lower in the groups whose energy intake from carbohydrate was <50% or ≥75%, compared to the others. The percentage of those who consumed less dairy products than the minimum amount was higher in the groups whose energy intake from carbohydrate was ≥65%, compared to the others. The possible explanation for this result is as follows. According to the previous studies, the dairy product intake tended to decrease as the level of energy intake from carbohydrate increased [9,42]. A study using the data from the 6th (2013–2015) KNHANES reported that a higher energy intake from carbohydrate was associated with less consumption of dairy products. For the dairy products, the ratio of the number of servings to the recommended number of servings tended to decrease as the level of energy intake from carbohydrate increased [9]. Another study of a Japanese population aged 40 to 70 years also reported that the dairy products intake significantly decreased as the level of energy intake from carbohydrate increased [42]. In summary, the food group intake was more inadequate in the groups whose energy intake from carbohydrate was <50% or ≥65%, compared to the others.

There are few studies which examine the association between various levels of energy intake from carbohydrate and risk factors such as metabolic syndrome, hypertriglyceridemia, low HDL-cholesterolemia, hypertension, or diabetes mellitus. In our study, no considerable difference was found in metabolic risk factors and diet quality between the groups of 50–55% and 55–65% energy intake from carbohydrate.

Our study has some limitations. First, we used a cross-sectional study design, which cannot draw any causal relationships. Second, the results might be different from subjects’ usual daily intake as we used the dietary data that were obtained using a 24-h recall method. The results presented in our study should be interpreted with consideration of these limitations.

## 5. Conclusions

The diet quality was much lower in the groups of <50% or ≥65% energy intake from carbohydrate, compared to the groups of 50–65% energy intake from carbohydrate. The odds ratio for low HDL-cholesterolemia or hypertension was significantly higher in the groups of <45% or ≥70% energy intake from carbohydrate, compared to the group of 60–65% energy intake from carbohydrate. We found no considerable difference in the diet quality and health conditions between the groups of 50–55% and 55–65% energy intake from carbohydrate. Therefore, this study suggests to expand the current AMDR for carbohydrate to 50–65% for Korean adults by adding 50–55% to the current range of 55–65%. Further cohort studies regarding the causal relationship between the level of energy intake from carbohydrate and health conditions could provide additional information for elucidating the optimal level of energy intake from carbohydrate for Korean adults.

## Figures and Tables

**Table 1:** The demographic characteristics of subjects according to the level of energy intake from carbohydrate.

Variable	<45% *(n* = 527)	45–50% (*n* = 459)	50–55% (*n* = 740)	55–60% (*n* = 1037)	60–65% (*n* = 1342)	65–70% (*n* = 1281)	70–75% (*n* = 1116)	≥75% (*n* = 1064)
	(%)							
Sex	*p* < 0.001							
Male	59.2	54.2	53.3	51.3	52.0	51.7	45.2	41.1
Female	40.8	45.8	46.7	48.7	48.0	48.3	54.8	58.9
Age (years)	*p* < 0.001							
19–29	34.4	33.1	31.2	25.2	22.2	15.0	15.2	6.2
30–49	48.6	49.0	49.8	51.2	49.9	49.7	40.9	35.4
50–64	17.0	17.8	19.0	23.6	28.0	35.3	43.9	58.4
Residential area	*p* = 0.001							
Urban	90.3	90.4	90.5	88.7	87.1	87.5	85.7	83.7
Rural	9.7	9.6	9.5	11.3	12.9	12.5	14.3	16.3
Household income level ^1^ (*n* = 7555)	*p* < 0.001							
Low	6.1	9.6	8.3	6.7	7.3	9.0	11.5	15.6
Medium-low	22.4	18.1	20.8	21.5	22.5	23.3	22.5	27.6
Medium-high	28.5	29.6	31.3	34.4	33.7	31.5	33.1	27.4
High	43.0	42.7	39.7	37.5	36.6	36.1	32.9	29.4
Education level ^1^ (*n* = 7224)	*p* < 0.001							
≤Elementary school graduate	1.5	2.1	2.0	3.6	4.2	5.9	8.5	18.0
Middle school graduate	4.1	3.6	5.1	5.4	4.7	9.4	9.8	14.4
High school graduate	40.4	35.6	40.4	36.6	38.3	37.8	38.0	35.4
≥College graduate	54.0	58.7	52.4	54.4	52.8	46.9	43.8	32.1
Employment status ^1^ (*n* = 7219)	*p* = 0.017							
Employed	27.9	29.5	33.9	29.0	28.6	28.5	30.9	35.9
Unemployed	72.1	70.5	66.1	71.0	71.4	71.5	69.1	64.1
Marriage status	*p* < 0.001							
Married	58.0	61.8	62.4	68.5	72.3	78.7	79.6	86.1
Unmarried	42.0	38.2	37.6	31.5	27.7	21.3	20.4	13.9
Number of family members	*p* < 0.001							
1	10.2	11.0	9.4	8.2	7.4	7.1	7.6	7.2
2	16.4	18.0	17.1	16.3	19.2	19.8	19.8	26.2
3	32.1	33.3	33.1	30.2	29.6	28.8	33.2	28.4
4	34.9	28.1	33.1	35.4	33.9	33.7	29.1	28.2
≥5	6.4	9.6	7.3	9.9	10.0	10.7	10.3	9.9

Notes: The data were analyzed using the complex sample module. *p*-value by Rao–Scott χ^2^ test. ^1^ Different from the total number of subjects due to the missing data. <45%: less than 45%, 45–50%: 45% to less than 50%, 50–55%: 50% to less than 55%, 55–60%: 55% to less than 60%, 60–65%: 60% to less than 65%, 65–70%: 65% to less than 70%, 70–75%: 70% to less than 75%, and ≥75%: 75% or more.

**Table 2:** Nutrient adequacy ratio (NAR) and mean adequacy ratio (MAR) of Korean adults according to the level of energy intake from carbohydrate.

	<45% *(n* = 527)	45–50% (*n* = 459)	50–55% (*n* = 740)	55–60% (*n* = 1037)	60–65% (*n* = 1342)	65–70% (*n* = 1281)	70–75% (*n* = 1116)	≥75% (*n* = 1064)	*p* ^1^
NAR	Mean ± SE								
Protein	0.98 ± 0.004 ^a,^ ^2^	0.97 ± 0.01 ^a^	0.95 ± 0.01 ^b^	0.96 ± 0.004 ^b^	0.93 ± 0.004 ^c^	0.90 ± 0.01 ^d^	0.86 ± 0.01 ^e^	0.77 ± 0.01 ^f^	<0.001
Vitamin A	0.56 ± 0.01 ^ab^	0.55 ± 0.01 ^ab^	0.55 ± 0.01 ^a^	0.50 ± 0.01 ^bc^	0.50 ± 0.01 ^b^	0.46 ± 0.01 ^cd^	0.43 ± 0.01 ^d^	0.36 ± 0.01 ^e^	<0.001
Thiamin	0.94 ± 0.01 ^bc^	0.95 ± 0.01 ^a^	0.94 ± 0.01 ^ab^	0.95 ± 0.005 ^a^	0.93 ± 0.005 ^ab^	0.92 ± 0.01 ^ab^	0.91 ± 0.01 ^ab^	0.89 ± 0.01 ^c^	<0.001
Riboflavin	0.94 ± 0.01 ^a^	0.92 ± 0.01 ^ab^	0.91 ± 0.01 ^ab^	0.91 ± 0.01 ^ab^	0.87 ± 0.01 ^b^	0.84 ± 0.01 ^c^	0.78 ± 0.01 ^d^	0.68 ± 0.01 ^e^	<0.001
Niacin	0.91 ± 0.01 ^a^	0.90 ± 0.01 ^a^	0.87 ± 0.01 ^ab^	0.87 ± 0.01 ^a^	0.83 ± 0.01 ^b^	0.80 ± 0.01 ^c^	0.75 ± 0.01 ^d^	0.69 ± 0.01 ^e^	<0.001
Vitamin C	0.49 ± 0.02 ^d^	0.53 ± 0.02 ^c^	0.56 ± 0.01 ^abc^	0.56 ± 0.01 ^bc^	0.57 ± 0.01 ^ab^	0.57 ± 0.01 ^abc^	0.58 ± 0.01 ^ab^	0.61 ± 0.02 ^a^	<0.001
Calcium	0.61 ± 0.01 ^d^	0.65 ± 0.01 ^abc^	0.65 ± 0.01 ^ab^	0.66 ± 0.01 ^ab^	0.64 ± 0.01 ^a^	0.62 ± 0.01 ^b^	0.58 ± 0.01 ^c^	0.50 ± 0.01 ^e^	<0.001
Phosphorus	0.98 ± 0.004 ^ab^	0.98 ± 0.004 ^a^	0.97 ± 0.004 ^ab^	0.97 ± 0.003 ^a^	0.97 ± 0.003 ^a^	0.95 ± 0.004 ^bc^	0.93 ± 0.01 ^c^	0.88 ± 0.01 ^d^	<0.001
Iron	0.89 ± 0.01 ^ab^	0.89 ± 0.01 ^ab^	0.88 ± 0.01 ^ab^	0.89 ± 0.01 ^ab^	0.89 ± 0.01 ^a^	0.88 ± 0.01 ^ab^	0.88 ± 0.01 ^ab^	0.87 ± 0.01 ^b^	0.022
MAR	0.81 ± 0.01 ^a^	0.82 ± 0.01 ^a^	0.81 ± 0.01 ^a^	0.81 ± 0.005 ^a^	0.79 ± 0.004 ^b^	0.77 ± 0.01 ^c^	0.74 ± 0.01 ^d^	0.69 ± 0.01 ^e^	<0.001

Notes: The data were analyzed using the complex sample module. <45%: less than 45%, 45–50%: 45% to less than 50%, 50–55%: 50% to less than 55%, 55–60%: 55% to less than 60%, 60–65%: 60% to less than 65%, 65–70%: 65% to less than 70%, 70–75%: 70% to less than 75%, and ≥75%: 75% or more. ^1^ For the NAR of protein and the MAR by ANCOVA with sex and age as covariates. For NARs of vitamins and minerals by ANCOVA with total energy intake in addition to sex and age. ^2^ Post-hoc test: Holm–Bonferroni, a > b > c > d > e > f.

**Table 3:** The dietary diversity score and its distribution of Korean adults according to the level of energy intake from carbohydrate.

	<45% (*n* = 527)	45–50% (*n* = 459)	50–55% (*n* = 740)	55–60% (*n* = 1037)	60–65% (*n* = 1342)	65–70% (*n* = 1281)	70–75% (*n* = 1116)	≥75% (*n* = 1064)	*p* ^2^
DDS ^1^	Mean ± SE								
	3.72 ± 0.03 ^d,3^	3.81 ± 0.04 ^bcd^	3.88 ± 0.04 ^abc^	3.97 ± 0.03 ^a^	3.96 ± 0.03 ^ab^	3.92 ± 0.03 ^abc^	3.90 ± 0.03 ^cd^	3.75 ± 0.03 ^e^	<0.001
DDS ^1^	**%**								
1	0.4	0.1	0.3	0.3	0.2	0.3	0.3	0.4	<0.001
2	1.5	1.1	2.8	1.6	0.8	1.5	2.8	5.4	
3	38.5	35.2	29.9	26.5	29.4	29.0	26.7	29.4	
4	44.8	44.8	42.9	44.0	42.1	44.4	46.4	48.6	
5	14.9	18.8	24.1	27.6	27.4	24.9	23.8	16.2	

Notes: The data were analyzed using the complex sample module. <45%: less than 45%, 45–50%: 45% to less than 50%, 50–55%: 50% to less than 55%, 55–60%: 55% to less than 60%, 60–65%: 60% to less than 65%, 65–70%: 65% to less than 70%, 70–75%: 70% to less than 75%, and ≥75%: 75% or more. ^1^ Dietary diversity score. ^2^ By Rao–Scott χ^2^ test or ANCOVA with sex and age as covariates. ^3^ Post-hoc test: Holm–Bonferroni, a > b > c > d > e.

**Table 4:** The food group intake patterns of Korean adults according to the level of energy intake from carbohydrate.

	<45% *(n* = 527)	45–50% (*n* = 459)	50–55% (*n* = 740)	55–60% (*n* = 1037)	60–65% (*n* = 1342)	65–70% (*n* = 1281)	70–75% (*n* = 1116)	≥75% (*n* = 1064)	*p* ^2^
DMGFV ^1^	%								
11111	14.9	18.8	24.1	27.6	27.4	24.9	23.8	16.2	<0.001
01111	24.8	22.0	23.2	26.5	25.7	31.0	34.6	41.8	
11101	18.6	21.9	19.3	17.2	15.8	12.0	10.2	3.5	
01101	35.9	32.9	28.1	25.6	27.8	27.2	25.1	19.9	
Others	5.8	4.4	5.3	3.1	3.3	4.9	6.3	18.6	

Note: The data were analyzed using the complex sample module. <45%: less than 45%, 45–50%: 45% to less than 50%, 50–55%: 50% to less than 55%, 55–60%: 55% to less than 60%, 60–65%: 60% to less than 65%, 65–70%: 65% to less than 70%, 70–75%: 70% to less than 75%, and ≥75%: 75% or more. ^1^ D: dairy products, M: meat/fish/eggs/legumes, G: grains, F: fruits, V: vegetables. In order of DMGFV, when more of each food group was consumed, 1 was assigned; otherwise, 0 was assigned. ^2^ By Rao–Scott χ^2^ test.

**Table 5:** The health characteristics of Korean adults according to the level of energy intake from carbohydrate. ^1.^

	<45% *(n* = 527)	45–50% (*n* = 459)	50–55% (*n* = 740)	55–60% (*n* = 1037)	60–65% (*n* = 1342)	65–70% (*n* = 1281)	70–75% (*n* = 1116)	≥75% (*n* = 1064)
	OR (95% CI) ^2^							
**Obesity**^3^ (*n* = 7559)	1.25 (0.97–1.61)	1.15 (0.88–1.50)	1.11 (0.90–1.37)	1.14 (0.93–1.40)	Reference	0.98 (0.80–1.21)	0.89 (0.73–1.08)	1.05 (0.85–1.29)
**Hypercholesterolemia**^3^ (*n* = 7205)	0.89 (0.65–1.21)	0.87 (0.60–1.26)	0.97 (0.72–1.31)	0.81 (0.62–1.06)	Reference	0.81 (0.64–1.03)	0.79 (0.61–1.02)	0.86 (0.68–1.10)
**Metabolic syndrome**^3,4^ (*n* = 7334)	1.06 (0.79–1.42)	0.98 (0.69–1.40)	1.14 (0.86–1.53)	0.88 (0.68–1.15)	Reference	0.99 (0.79–1.24)	0.98 (0.76–1.27)	1.10 (0.86–1.40)
**Risk factors of metabolic syndrome**^3,5^ (*n* = 7334)								
High waist circumference	1.16 (0.88–1.52)	1.12 (0.84–1.50)	1.16 (0.91–1.49)	1.09 (0.87–1.36)	Reference	0.98 (0.79–1.22)	1.05 (0.84–1.32)	1.05 (0.83–1.32)
Hypertriglyceridemia	1.03 (0.81–1.30)	1.23 (0.92–1.63)	1.13 (0.90–1.44)	1.07 (0.86–1.34)	Reference	1.10 (0.91–1.33)	1.00 (0.81–1.24)	1.14 (0.92–1.40)
Low HDL-cholesterolemia	1.00 (0.78–1.28)	0.89 (0.66–1.19)	1.07 (0.85–1.35)	1.13 (0.91–1.39)	Reference	1.18 (0.97–1.43)	1.20 (0.98–1.47)	1.37 (1.10–1.70) **
Hypertension	1.48 (1.10–2.00) **	1.20 (0.89–1.61)	1.16 (0.91–1.48)	0.97 (0.77–1.22)	Reference	0.96 (0.79–1.18)	1.25 (1.00–1.55) *	1.10 (0.87–1.38)
Impaired fasting glucose	0.96 (0.66–1.40)	0.94 (0.63–1.40)	1.05 (0.73–1.50)	0.83 (0.62–1.12)	Reference	0.99 (0.76–1.29)	1.01 (0.77–1.31)	1.10 (0.86–1.41)

* *p* < 0.05, ** *p* < 0.005. Note: The data were analyzed using the complex sample module. <45%: less than 45%, 45–50%: 45% to less than 50%, 50–55%: 50% to less than 55%, 55–60%: 55% to less than 60%, 60–65%: 60% to less than 65%, 65–70%: 65% to less than 70%, 70–75%: 70% to less than 75%, and ≥75%: 75% or more. ^1^ Result of multiple logistic regression analysis adjusted for sex, age, and total energy intake. ^2^ OR: odds ratio, CI: confidence interval. ^3^ Different from the total number of subjects due to missing data. ^4^ Having three or more risk factors of metabolic syndrome. ^5^ High waist circumference (greater than or equal to 90 cm for men and greater than or equal to 85 cm for women), hypertriglyceridemia (greater than or equal to 150 mg/dL or in the treatment of related medication), low HDL-cholesterolemia (less than 40 mg/dL for men and less than 50 mg/dL for women), hypertension (systolic blood pressure greater than or equal to 130 mmHg, diastolic blood pressure greater than or equal to 85 mmHg, or in the treatment of related medication), and impaired fasting glucose (greater than or equal to 110 mg/dL or in the treatment of related medication).

## Supplementary Materials

The following are available online at https://www.mdpi.com/2072-6643/12/2/429/s1, Table S1: The nutrient intake of Korean adults according to the level of energy intake from carbohydrate, Table S2: The food group intake of Korean adults according to the level of energy intake from carbohydrate, Table S3: The biochemical indices of Korean adults according to the level of energy intake from carbohydrate.
