# Supplementary material for: Dietary and Health Characteristics of Korean Adults According to the Level of Energy Intake from Carbohydrate: Analysis of the 7th (2016–2017) Korea National Health and Nutrition Examination Survey Data
Source: Nutrients. 2020 Feb 7;12(2):429. doi: 10.3390/nu12020429 (PMC7071296; doi:10.3390/nu12020429)
Supplement: Supplementary file 1 [file nutrients-12-00429-s001.pdf]

Table S1. The energy and nutrient intake of Korean adults according to the level of energy intake from carbohydrate.

	<45% than 45% (n = 527)	45–50% 50% (n = 459)	50–55%55% (n = 740)	55–60% 60% (n = 1037)	60–65%65% (n = 1342)	65–70% 70% (n = 1281)	70–75% 75% (n = 1116)	≥75% (n = 1064)	p [1]
					Mean ± SE				
Energy (kcal)	2292 ± 45 [a 2]	2156 ± 42 [b]	2090 ± 34 [b]	2053 ± 28 [b]	1932 ± 22 [c]	1898 ± 23 [c]	1778 ± 25 [d]	1684 ± 26 [e]	<0.001
Protein (g)	117.6 ± 2.7 [a]	99.7 ± 2.2 [b]	91.4 ± 1.8 [c]	82.7 ± 1.2 [d]	73.9 ± 0.9 [e]	67.3 ± 0.9 [f]	57.6 ± 0.8 [g]	47.3 ± 0.8 [h]	<0.001
Fat (g)	105.9 ± 2.6 [a]	80.7 ± 1.8 [b]	69.6 ± 1.2 [c]	59.9 ± 0.9 [d]	47.4 ± 0.6 [e]	38.9 ± 0.5 [f]	29.0 ± 0.4 [g]	17.7 ± 0.3 [h]	<0.001
Vitamin A (μg RAE)	543 ± 44 [a]	452 ± 24 [ab]	449 ± 16 [a]	408 ± 12 [abc]	416 ± 24 [ab]	359 ± 11 [bc]	328 ± 10 [c]	267 ± 16 [d]	<0.001
Thiamin (mg)	2.38 ± 0.08 [a]	2.01 ± 0.05 [b]	1.95 ± 0.04 [b]	1.87 ± 0.03 [b]	1.66 ± 0.02 [c]	1.63 ± 0.03 [c]	1.52 ± 0.02 [c]	1.40 ± 0.02 [d]	<0.001
Riboflavin (mg)	2.09 ± 0.05 [a]	1.83 ± 0.05 [b]	1.78 ± 0.03 [b]	1.70 ± 0.02 [b]	1.55 ± 0.02 [c]	1.44 ± 0.02 [d]	1.23 ± 0.02 [e]	0.99 ± 0.02 [f]	<0.001
Niacin (mg)	23.3 ± 0.7 [a]	19.9 ± 0.5 [b]	19.3 ± 0.4 [b]	17.1 ± 0.3 [c]	15.0 ± 0.2 [d]	14.1 ± 0.2 [e]	12.5 ± 0.2 [f]	11.0 ± 0.2 [g]	<0.001
Vitamin C (mg)	66 ± 4 [d]	69 ± 3 [d]	82 ± 4 [bc]	80 ± 3 [c]	80 ± 3 [bc]	86 ± 4 [bc]	89 ± 4 [ab]	101 ± 4 [a]	<0.001
Calcium (mg)	529 ± 17 [b]	545 ± 17 [ab]	536 ± 11 [ab]	553 ± 11 [a]	532 ± 9 [a]	502 ± 8 [b]	463 ± 9 [b]	392 ± 7 [c]	<0.001
Iron (mg)	16.4 ± 0.5	16.7 ± 0.5	16.7 ± 0.5	15.7 ± 0.3	15.2 ± 0.3	14.8 ± 0.3	14.7 ± 0.4	13.5 ± 0.3	0.062

Note: The data were analyzed using the complex sample module. <45%: less than 45%, 45–50%: 45% to less than 50%, 50–55%: 50% to less than 55%, 55–60%: 55% to less than 60%, 60–65%: 60% to less than 65%, 65–70%: 65% to less than 70%, 70–75%: 70% to less than 75%, and ≥75%: 75% or more. [1] For energy by ANCOVA with sex and age as covariates. For the rest of nutrients by ANCOVA with total energy intake in addition to sex and age. [2] Post-hoc test: Holm–Bonferroni, a > b > c > d > e > f > g > h.

Table S2. The food group intake of Korean adults according to the level of energy intake from carbohydrate.

	**<45% (n=527)**	**45–50% (n=459)**	**50–55% (n=740)**	**55–60% (n=1037)**	**60–65% (n=1342)**	**65–70% (n=1281)**	**70–75% (n=1116)**	**≥75% (n= 1064)**	p [1]
	Mean ± SE								
Grains	205 ± 6 [h 2]	275 ± 9 [g]	301 ± 7 [f]	329 ± 6 [e]	325 ± 4 [d]	348 ± 5 [c]	361 ± 7 [b]	372 ± 8 [a]	<0.001
Meat·fish·eggs·legumes	511 ± 13 [a]	422 ± 15 [b]	377 ± 10 [c]	325 ± 7 [d]	290 ± 6 [e]	265 ± 7 [f]	217 ± 7 [g]	151 ± 6 [h]	<0.001
Vegetables	321 ± 10 [c]	337 ± 14 [ab]	344 ± 10 [ab]	335 ± 9 [ab]	329 ± 6 [b]	364 ± 8 [a]	354 ± 8 [ab]	345 ± 10 [ab]	<0.001
Fruits	101 ± 8 [g]	109 ± 9 [fg]	129 ± 8 [ef]	161 ± 9 [de]	172 ± 8 [cd]	191 ± 9 [bc]	207 ± 10 [b]	295 ± 13 [a]	<0.001
Dairy products	90 ± 9 [bcde]	114 ± 9 [abc]	102 ± 6 [bcd]	111 ± 6 [ab]	104 ± 5 [abc]	83 ± 4 [cd]	66 ± 4 [d]	43 ± 3 [e]	<0.001

Note: The data were analyzed using the complex sample module. <45%: less than 45%, 45–50%: 45% to less than 50%, 50–55%: 50% to less than 55%, 55–60%: 55% to less than 60%, 60–65%: 60% to less than 65%, 65–70%: 65% to less than 70%, 70–75%: 70% to less than 75%, and ≥75%: 75% or more. [1] By ANCOVA with sex, age and the total energy intake as covariates. [2] Post-hoc test: Holm–Bonferroni, a > b > c > d > e > f > g > h.

Table S3. The biochemical indices of Korean adults according to the level of energy intake from carbohydrate.

	**<45% (*n* = 527)**	**45–50% (*n* = 459)**	**50–55% (*n* = 740)**	**55–60% (*n* = 1037)**	**60–65% (*n* = 1342)**	**65–70% (*n* = 1281)**	**70–75% (*n* = 1116)**	**≥75% (*n* = 1064)**	*p* [1]
	Mean ± SE								
Systolic blood pressure (mmHg)	114.3 ± 0.6	114.4 ± 0.7	113.9 ± 0.4	113.7 ± 0.5	115.1 ± 0.4	116.0 ± 0.4	116.6 ± 0.5	117.8 ± 0.6	0.454
Diastolic blood pressure (mmHg)	76.3 ± 0.5	76.4 ± 0.5	76.2 ± 0.4	76.0 ± 0.3	76.3 ± 0.3	76.8 ± 0.3	77.2 ± 0.3	76.4 ± 0.3	0.094
BMI (kg/m²)	24.3 ± 0.1	23.7 ± 0.2	23.9 ± 0.1	23.8 ± 0.1	23.7 ± 0.1	23.9 ± 0.1	23.7 ± 0.1	23.9 ± 0.1	0.166
Waist circumference (cm)	82.2 ± 0.5	81.2 ± 0.5	81.7 ± 0.4	81.4 ± 0.3	81.1 ± 0.3	81.9 ± 0.3	81.2 ± 0.3	81.9 ± 0.4	0.273
Triglyceride (mg/100mL)	139 ± 7	141 ± 8	137 ± 6	136 ± 4	135 ± 3	145 ± 4	135 ± 4	143 ± 3	0.897
Total cholesterol (mg/100mL)	191 ± 1 [a2]	193 ± 1 [a]	191 ± 1 [ab]	194 ± 1 [a]	194 ± 1 [a]	196 ± 1 [a]	194 ± 1 [ab]	193 ± 1 [b]	0.005
HDL-cholesterol (mg/100mL)	53.0 ± 0.5 [a]	52.9 ± 0.6 [ab]	52.0 ± 0.5 [abc]	52.2 ± 0.4 [ab]	52.4 ± 0.4 [ab]	51.1 ± 0.3 [bc]	51.3 ± 0.4 [bc]	50.3 ± 0.4 [c]	<0.001
LDL-cholesterol (mg/100mL)	117 ± 4	121 ± 4	119 ± 3	115 ± 2	123 ± 2	120 ± 2	122 ± 3	115 ± 2	0.403
Fasting glucose (mg/100mL)	95.5 ± 0.9	96.4 ± 0.8	95.8 ± 0.6	96.2 ± 0.6	98.8 ± 0.8	98.0 ± 0.6	98.1 ± 0.6	102.6 ± 1.0	0.222

Note: The data was analyzed using the complex sample module. <45%: less than 45%, 45–50%: 45% to less than 50%, 50–55%: 50% to less than 55%, 55–60%: 55% to less than 60%, 60–65%: 60% to less than 65%, 65–70%: 65% to less than 70%, 70–75%: 70% to less than 75%, and ≥75%: 75% or more. [1] By ANCOVA with sex and age as covariates. [2] Post-hoc test: Holm–Bonferroni, a> b > c.
